# Real-world effectiveness of liraglutide versus dulaglutide in Japanese patients with type 2 diabetes: a retrospective study

**DOI:** 10.1038/s41598-021-04149-z

**Published:** 2022-01-07

**Authors:** Kenichi Tanaka, Yosuke Okada, Akemi Tokutsu, Yoshiya Tanaka

**Affiliations:** First Department of Internal Medicine, School of Medicine, University of Occupational and Environmental Health, Japan, 1-1 Iseigaoka, Yahatanishi-ku, Kitakyushu, 807-8555 Japan

**Keywords:** Endocrinology, Endocrine system and metabolic diseases

## Abstract

Real-world data comparing the effectiveness of various glucagon-like peptide 1 receptor agonists (GLP-1 RAs) in type 2 diabetes mellitus (T2DM) are limited. We investigated the clinical effectiveness of liraglutide and dulaglutide in Japanese T2DM in a real-world setting. This retrospective study included 179 patients with T2DM who were treated with GLP-1 RA for at least 12 months (liraglutide, n = 97; dulaglutide, n = 82). We used stabilized propensity score-based inverse probability of treatment weighting (IPTW) to reduce selection bias and confounding by observed covariates. Changes in glycated hemoglobin (HbA1c) at the end of the 12-month treatment were evaluated. After adjustment by stabilized propensity score-based IPTW, no significant differences were observed in patient characteristics between the liraglutide and dulaglutide groups. HbA1c was significantly lower at 12 months in both groups (liraglutide, 8.9 to 7.4%; dulaglutide, 8.7 to 7.5%). Multivariate linear regression analysis showed no differences in the extent of changes in HbA1c at 12 months between the two agents. High baseline HbA1c, the addition of GLP-1 RA treatment modality, and in-hospital initiation of GLP-1 RA treatment were identified as significant contributing factors to HbA1c reduction. The effects of liraglutide and dulaglutide on lowering HbA1c levels at 12 months were comparable in a real-world setting.

## Introduction

Glucagon-like peptide-1 (GLP-1), an incretin hormone secreted by L-cells in the distal ileum and colon^1^, has various effects, including glucose-dependent enhancement of insulin secretion^2^, inhibition of glucagon secretion^3^, appetite suppression^4^, and suppression of gastric emptying^5^. Various GLP-1 receptor agonists (GLP-1 RAs) are available in the market at present. GLP-1 RAs can effectively lower blood glucose levels and facilitate weight loss with a low risk of hypoglycemia; hence, they are commonly used in patients with type 2 diabetes mellitus (T2DM). Furthermore, some GLP-1 RAs are reported to reduce the risk of 3-point major adverse cardiovascular events (MACE)^6, 7^, and a meta-analysis of four large-scale clinical studies that assessed GLP-1 RAs concluded that these agents can reduce the risk of cardiovascular events and all-cause mortality^8^.

Controlled clinical trials have demonstrated that GLP-1 RAs have distinct effects in terms of lowering glycated hemoglobin (HbA1c) levels and facilitating weight loss, as well as adverse reactions, such as nausea, vomiting, and diarrhea^8, 9^. In Japan, dulaglutide and liraglutide are the most widely used GLP-1 RAs in routine clinical practice^10^. In a randomized controlled trial (RCT), dulaglutide (up to 1.5 mg/week) was reported to be non-inferior to liraglutide (up to 1.8 mg/day)^11^. In a Japanese comparative study of these agents used at different doses, HbA1c-lowering effects after 52 weeks of treatment were greater with dulaglutide (up to 0.75 mg/week) than liraglutide (up to 0.9 mg/day)^12^. However, the above study evaluated patients who were not taking antidiabetic drugs or had discontinued antidiabetic monotherapy, thereby differing greatly from real-world clinical situations. In a real-world setting, only a few studies have investigated the effectiveness of liraglutide and dulaglutide in patients with T2DM, including those treated concomitantly with more than one oral hypoglycemic agent and insulin. To establish real-world evidence, we retrospectively examined changes in HbA1c after 12 months of GLP-1 RA treatment and compared liraglutide and dulaglutide. In this study, we used stabilized propensity score-based inverse probability of treatment weighting (IPTW) in order to reduce selection bias and confounding by observed covariates.

## Results

### Patient demographics after adjustment by stabilized propensity score-based IPTW

The selection of GLP-1 RA for treatment of these patients, and specifically with liraglutide and dulaglutide was based on the clinical assessment and judgment by the attending physicians. Table 1 (left-side) summarizes the clinical characteristics of the study patients before adjustment. Ninety-seven patients treated with liraglutide and 82 patients treated with dulaglutide at least 12 months. The average liraglutide dose were administered at 0.89 mg/day (SD: 0.26) and dulaglutide dose were 0.75 mg/week at 12 months, respectively. Compared with patients of the dulaglutide group, those of the liraglutide group were significantly younger, had shorter duration of T2DM, higher BMI, and used fewer classes of oral glucose-lowering agents. Higher prevalence of dementia was noted in the dulaglutide group. Next, we calculated the IPTW using the stabilized propensity scores to reduce selection bias and confounding by observed covariates and adjusted the patient characteristics. The adjusted patient characteristics are shown in Table 1 (right side). Ninety-seven and 72 patients were treated with liraglutide and dulaglutide, respectively. The distribution of the patients’ characteristics between the groups was balanced based on the small standardized mean difference and the insignificant P values.

**Table 1:** Baseline characteristics of the study patients before and after IPTW.

	Before IPTW				After IPTW			
	Liraglutide	Dulaglutide	P value	SMD	Liraglutide	Dulaglutide	P value	SMD
n	97	82			97	72		
Age (years)	60.7 (12.5)	68.6 (10.1)	< 0.001	0.70	63.3 (10.9)	64.7 (11.3)	0.419	0.13
Sex (men/women)	51/46	40/42	0.714	0.08	49/48	36/36	1.000	0.01
Duration of diabetes (year)	13.9 (8.5)	17.4 (9.8)	0.011	0.38	14.5 (9.6)	16.5 (10.7)	0.193	0.20
Body weight (kg)	71.5 (17.5)	59.3 (11.9)	< 0.001	0.82	66.0 (16.6)	62.1 (12.1)	0.096	0.27
Body mass index (kg/m^2^)	27.8 (6.0)	24.1 (4.6)	< 0.001	0.69	26.0 (5.4)	24.6 (3.8)	0.096	0.29
Systolic blood pressure (mmHg)	132.3 (19.8)	127.7 (16.8)	0.146	0.25	130.3 (19.1)	128.8 (17.2)	0.609	0.08
Diastolic blood pressure (mmHg)	75.3 (12.6)	71.5 (10.5)	0.085	0.33	74.4 (11.8)	74.0 (10.5)	0.810	0.04
PG (mg/dL)	180.5 (66.6)	177.2 (72.0)	0.752	0.05	177.6 (58.9)	175.2 (66.0)	0.799	0.04
HbA1c (%)	8.9 (1.7)	8.8 (1.7)	0.746	0.08	8.9 (1.5)	8.7 (1.7)	0.368	0.10
AST (U/L)	27.1 (19.6)	24.2 (11.9)	0.248	0.18	23.4 (16.4)	23.3 (10.9)	0.959	0.01
ALT (U/L)	31.5 (31.4)	24.3 (17.4)	0.065	0.28	26.7 (26.2)	25.2 (19.9)	0.683	0.06
GGT (U/L)	46.4 (37.6)	40.1 (66.8)	0.441	0.12	42.2 (36.6)	38.0 (53.5)	0.543	0.09
eGFR (mL/min/1.73 m^2^)	65.4 (30.2)	59.5 (22.8)	0.154	0.22	63.4 (29.8)	60.4 (25.2)	0.491	0.11
Hypertension (%)	80 (82.5)	56 (68.3)	0.027	0.33	75 (77.3)	48 (66.7)	0.124	0.24
Dyslipidemia (%)	87 (89.7)	66 (80.5)	0.082	0.26	85 (87.6)	61 (84.7)	0.586	0.08
Antihypertensive agents (%)	77 (79.4)	52 (63.4)	0.018	0.36	73 (75.3)	46 (63.9)	0.109	0.25
Antilipidemic agents (%)	73 (75.3)	53 (64.6)	0.121	0.23	64 (66.0)	43 (59.7)	0.404	0.13
**Glucose-lowering agents used**								
None (%)	20 (20.6)	5 (6.1)	0.005	0.44	14 (14.4)	11 (15.3)	0.857	0.03
DPP-4 inhibitors (%)	61 (62.9)	67 (81.7)	0.005	0.43	67 (69.1)	53 (73.6)	0.520	0.10
Sulfonylurea (%)	12 (12.4)	16 (19.5)	0.190	0.19	21 (21.6)	11 (15.3)	0.296	0.16
Glinide (%)	13 (13.4)	27 (32.9)	0.002	0.48	14 (14.4)	19 (26.4)	0.053	0.29
Biguanides (%)	43 (44.3)	32 (39.0)	0.473	0.11	37 (38.1)	27 (37.5)	0.932	0.01
Thiazolidine (%)	23 (23.7)	18 (22.0)	0.780	0.04	20 (20.6)	19 (26.4)	0.379	0.14
α-glucosidase inhibitors (%)	21 (21.6)	16 (19.5)	0.725	0.05	20 (20.6)	12 (16.7)	0.517	0.10
SGLT-2 inhibitors (%)	8 (8.2)	15 (18.3)	0.045	0.30	10 (10.3)	9 (12.5)	0.639	0.07
Insulin (%)	55 (56.7)	35 (41.2)	0.062	0.31	48 (49.5)	35 (48.6)	0.911	0.02
Number of oral antidiabetic agent classes	1.9 (1.2)	2.3 (1.1)	0.007	0.01	2.0 (1.1)	2.1 (1.2)	0.664	0.01
Retinopathy (%)	50 (51.5)	41 (50.0)	0.837	0.03	60 (61.9)	35 (48.6)	0.102	0.27
Nephropathy (%)	50 (51.5)	37 (45.1)	0.392	0.13	49 (50.5)	31 (43.1)	0.337	0.15
Peripheral neuropathy (%)	62 (63.9)	50 (61.0)	0.685	0.06	70 (72.2)	46 (63.9)	0.252	0.18
Coronary heart disease (%)	19 (19.6)	12 (14.6)	0.845	0.13	19 (19.4)	11 (15.3)	0.487	0.11
Cerebrovascular disease (%)	14 (14.4)	11 (13.4)	0.383	0.03	16 (16.5)	9 (12.5)	0.469	0.11
Dementia (%)	4 (4.1)	14 (17.1)	0.004	0.43	15 (15.5)	8 (11.1)	0.414	0.13
**GLP-1RA treatment modality (%)**^**†**^			0.010	0.47			0.602	0.13
Add-on	16 (16.5)	9 (11.0)			11 (11.2)	11 (15.3)		
Switch	37 (38.1)	50 (61.0)			54 (55.1)	41 (56.9)		
Reduce	44 (45.1)	23 (28.0)			33 (33.7)	20 (27.8)		
Inpatient initiation of GLP-1 RA treatment	36 (37.1)	26 (31.7)	0.449	0.11	39 (40.2)	24 (33.3)	0.361	0.14

Data are mean (standard deviation), or n (%).

Comparisons between liraglutide and dulaglutide by the Student’s *t*-test or Wilcoxon rank-sum test.

*PG* plasma glucose, *HbA1c* glycated hemoglobin, *AST* aspartate transaminase, *ALT* alanine transaminase, *GGT* gamma-glutamyl transferase, *eGFR* estimated glomerular filtration rate, *DPP-4* dipeptidyl peptidase-4, *SGLT-2* sodium-glucose cotransporter 2, *GLP-1 RA* glucagon-like peptide-1 receptor agonist, *IPTW* inverse probability of treatment weighting, *SMD* standardized mean difference.

Categorical values were tested by -χ^2^ test. P values are for differences between the two groups.

^†^Baseline drug adjustments at liraglutide or dulaglutide initiation were categorized as follows: add-on, when the number of classes of glucose-lowering agents increased; switch, when the number of classes of glucose-lowering agents remained unchanged; reduce, when the number of classes of glucose-lowering agents decreased.

### Efficacy

**Figure 1:**
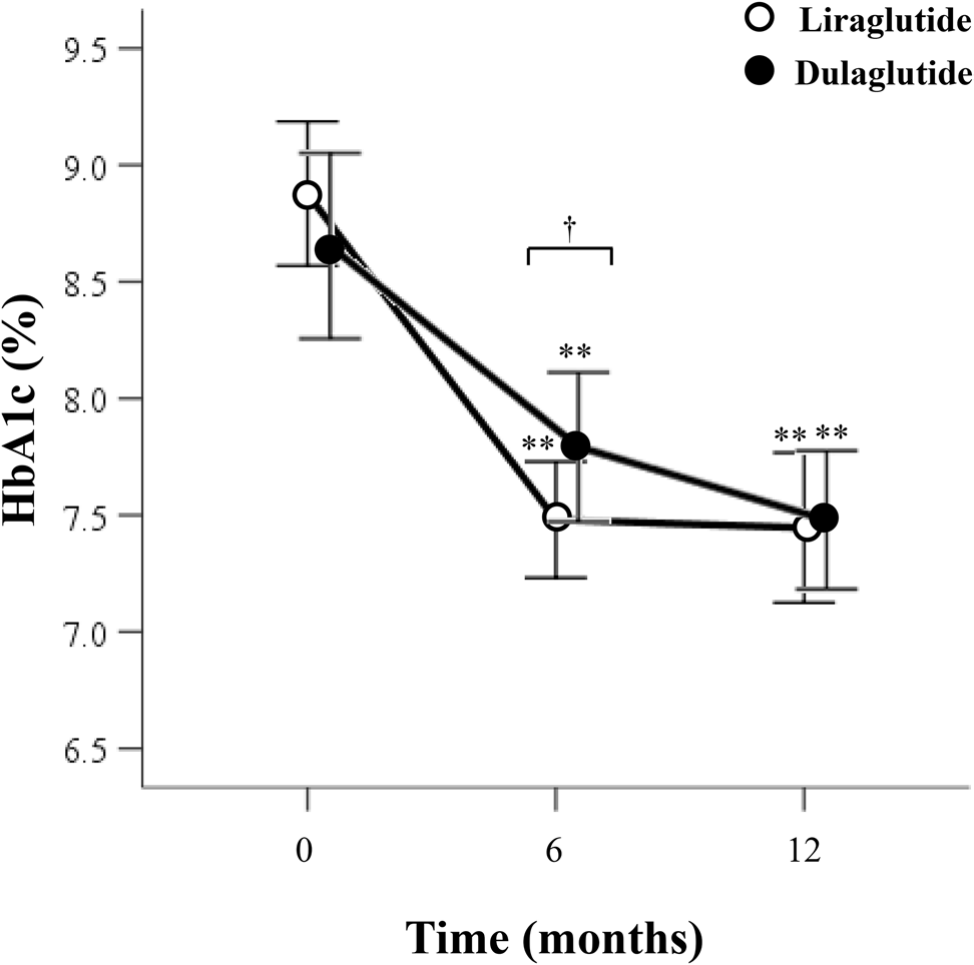
HbAlc levels at baseline and 6 and 12 months of treatment after adjustment by stabilized propensity score-based inverse probability of treatment weighting. Data are mean (95% CI). **P < 0.01 *vs*. baseline, by Wilcoxon signed-rank test. ^†^P < 0.05 for between the two groups, by Wilcoxon rank-sum test. *HbA1c* glycated hemoglobin.

**Figure 2:**
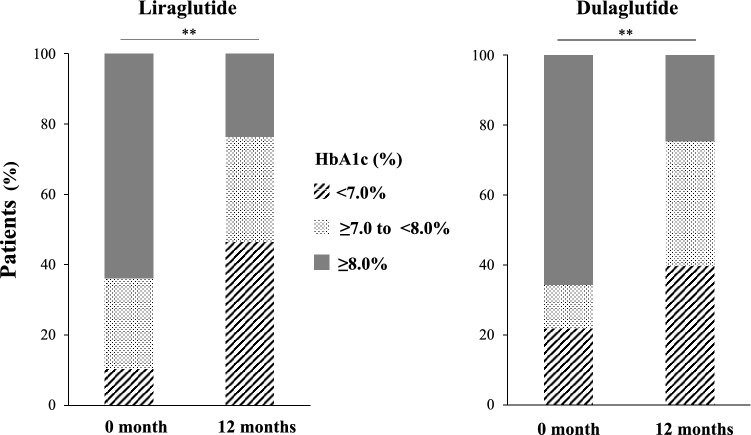
HbA1c target achievement at baseline and 12 months after adjustment by stabilized propensity score-based inverse probability of treatment weighting. **P < 0.01, by McNemar’s test. *HbA1c* glycated hemoglobin.

Figure 1 summarizes the effects of each treatment on HbA1c in the two groups after adjustment by stabilized propensity score-based IPTW. HbA1c was significantly lower at 12 months in both groups, relative to the respective value at baseline (before treatment), from 8.9 (SD: 1.5) to 7.4% (SD: 1.6) with liraglutide treatment and from 8.7 (SD: 1.7) to 7.5% (SD: 1.3) with dulaglutide therapy (Fig. 1). The change in HbA1c level was significantly different at 6 months of treatment (liraglutide *vs*. dulaglutide: − 1.4% [SD: 1.8] *vs*. − 1.2% [SD: 1.9], P = 0.016), but not at 12 months (− 1.4% [SD: 1.8] *vs*. − 1.2% [SD: 1.9], P = 0.100). This result was similar to the data before adjustment (Fig. S1). The percentage of patients who achieved HbA1c < 7% at 12 months was significant, compared to that at baseline, in both the liraglutide and dulaglutide groups (liraglutide: 10.3% to 46.4%; dulaglutide: 21.9% to 39.7%, Fig. 2). Similarly, the percentage of patients with HbA1c ≥ 8% significantly decreased in both groups, with no difference observed in the distribution of HbA1c at 12 months between the two groups.

Table 2 shows the effect of 12-month treatment with liraglutide and dulaglutide on various parameters, relative to the baseline. Body weight decreased significantly in both groups, though the reduction over the 12-month period differed significantly between the two groups (liraglutide *vs*. dulaglutide: − 2.7 kg [SD: 4.5] *vs*. − 1.2 kg [SD: 3.2], P = 0.005). The total and bolus insulin dose also decreased significantly in both groups, but no significant differences were observed between the two groups. eGFR was significantly lower in the liraglutide group, but no significant differences were observed between the two groups.

**Table 2:** Effects of treatment on various clinical and laboratory parameters after adjustment by stabilized propensity score-based IPTW.

	Liraglutide (n = 97)				Dulaglutide (n = 72)				
	0 month	12 months	Change	P*	0 month	12 months	Change	P*	P**
Body weight	66.0 (16.6)	63.2 (16.4)	− 2.7 (4.5)	< 0.001	62.1 (12.1)	61.1 (12.4)	− 1.0 (3.2)	0.012	0.005
Systolic blood pressure (mmHg)	130.3 (19.1)	129.4 (17.8)	− 0.9 (20.2)	0.679	128.8 (17.2)	126.4 (15.5)	− 2.4 (16.1)	0.227	0.616
Diastolic blood pressure (mmHg)	74.4 (11.8)	73.2 (11.5)	− 1.2 (11.2)	0.303	74.0 (10.5)	72.6 (10.0)	− 1.4 (10.7)	0.286	0.928
PG (mg/dL)	177.6 (58.9)	178.4 (65.6)	0.8 (71.8)	0.917	175.2 (66.0)	156.3 (64.4)	− 18.8 (70.4)	0.026	0.078
AST (U/L)	23.4 (16.5)	23.5 (12.1)	0.1 (16.5)	0.954	23.4 (10.9)	26.9 (15.7)	3.6 (15.3)	0.053	0.166
ALT (U/L)	26.7 (26.2)	23.8 (20.9)	− 2.9 (24.2)	0.242	25.3 (20.0)	28.3 (19.5)	3.0 (19.4)	0.202	0.094
GGT (U/L)	39.6 (32.1)	36.0 (30.4)	− 3.6 (20.5)	0.093	38.8 (56.7)	40.5 (71.8)	1.7 (25.0)	0.597	0.121
eGFR (mL/min/1.73 m^2^)	63.4 (29.8)	60.6 (27.3)	− 2.8 (10.9)	0.013	60.4 (25.2)	59.0 (26.9)	− 1.4 (10.5)	0.262	0.407
Insulin treatment (%)	48 (49.5)	42 (43.3)	–	0.500	35 (48.6)	26 (36.1)	–	0.031	
Total insulin dose (units/day)	15.5 (15.6)	9.7 (9.4)	− 5.8 (15.8)	0.005	14.9 (13.0)	10.6 (13.7)	− 4.3 (10.6)	0.015	0.607
Basal insulin dose (units/day)	10.3 (9.6)	9.0 (7.9)	− 1.3 (10.6)	0.362	10.2 (7.3)	9.2 (10.0)	− 1.0 (6.7)	0.340	0.906
Bolus insulin dose (units/day)	10.8 (9.3)	2.2 (4.2)	− 8.6 (9.6)	< 0.001	11.7 (7.4)	3.8 (8.0)	− 7.8 (9.7)	0.005	0.798
Augmentation (%)	–	15 (15.5)			–	17 (23.6)			0.196

Data are mean (standard deviation), or n (%).

*PG* plasma glucose, *AST* aspartate transaminase, *ALT* alanine transaminase, *GGT* gamma-glutamyl transferase, *eGFR* estimated glomerular filtration rate, *IPTW* inverse probability of treatment weighting.

*P-value for comparisons between 0 and 12 months, by the paired t-test or Wilcoxon signed-rank test.

**P-value for difference in changes from baseline between liraglutide and dulaglutide, by Student's t-test or Wilcoxon rank sum test.

We employed univariate and multivariate linear regression analysis to determine the factors that contributed to the observed changes in HbA1c after 12 months of treatment (Table 3). Multivariate linear regression analysis showed that the change in HbA1c after 12 months of treatment correlated positively with reducing or switching to GLP-1 RA treatment, and negatively with baseline HbA1c and inpatient initiation of GLP-1 RA treatment, but no difference was detected between the two types of GLP-1 RAs.

**Table 3:** Multivariate linear regression analysis with changes in HbA1c at 12 months as the dependent variable in patients with type 2 diabetes mellitus after adjustment by stabilized propensity score-based IPTW.

Variables	Univariate		Multivariate	
	β (95% CI)	P-value	β (95% CI)	P-value
**GLP-1 RA treatment**				
Liraglutide (reference)				
Dulaglutide	0.070 (− 0.300, 0.812)	0.365	0.012 (− 0.356, 0.444)	0.828
Duration of diabetes	0.230 (0.015, 0.068)	0.003	0.400 (− 0.013, 0.032)	0.400
PG at 0 M	− 0.202 (− 0.010, − 0.002)	0.008	− 0.104 (− 0.007, 0.001)	0.097
HbA1c at 0 M	− 0.641 (− 0.857, − 0.592)	< 0.001	− 0.418 (− 0.630, − 0.317)	< 0.001
Hypertension	0.191 (0.166, 1.381)	0.013	< 0.001 (− 0.520, 0.519)	0.999
Nephropathy	0.212 (0.229, 1.308)	0.006	0.049 (− 0.252, 0.605)	0.417
Coronary heart disease	0.193 (0.203, 1.627)	0.012	0.102 (− 0.071, 1.043)	0.087
Dementia	− 0.322 (− 2.474, − 0.941)	0.001	− 0.091 (− 1.135, 0.174)	0.149
Number of oral antidiabetic agent classes at 0 M	0.185 (0.057, 0.540)	0.001	0.108 (− 0.024, 0.375)	0.084
Insulin treatment at 0 M	0.337 (0.698, 1.737)	< 0.001	0.114 (− 0.052, 0.877)	0.081
**GLP-1RA treatment modality **				
Add-on (reference)				
Reduce	0.493 (1.104, 2.679)	< 0.001	0.245 (0.302, 1.596)	0.004
Switch	0.402 (0.718, 2.212)	< 0.001	0.224 (0.193, 1.448)	0.011
**Initiation of GLP-1 RA treatment**				
Outpatient (reference)				
Inpatient	− 0.504 (− 2.376, − 1.390)	< 0.001	− 0.217 (− 1.358, − 0.267)	0.004
			R^2^ = 0.530	
			P < 0.001	

*PG* plasma glucose, *HbA1c* glycated hemoglobin, *GLP-1 RA* glucagon-like peptide-1 receptor agonist, *M* month, *IPTW* inverse probability of treatment weighting.

Factors with P < 0.05 on univariate linear regression analysis and GLP-1 RA treatment were entered in this multivariate linear regression analysis.

Finally, we determined the factors that contributed to the observed changes in HbA1c after 12 months of treatment using univariate and multivariate analyses in non-adjusted data (Supplemental Tables S1, S2). Multivariate analysis identified baseline HbA1c, baseline plasma glucose, and inpatient initiation of GLP-1 RA treatment as significant independent factors that contributed to the change in HbA1c over the 12-month liraglutide treatment. Similarly, baseline HbA1c and GLP-1 RA treatment modality were identified as significant factors that contributed to the observed changes in HbA1c over the 12-month dulaglutide treatment.

### Safety

Adverse reactions were observed in 27 patients (27.8%) of the liraglutide group and 20 patients (24.4%) of the dulaglutide group (P = 0.602) in non-adjusted data. Only mild adverse reactions were observed in both groups. The reported adverse reactions in the liraglutide group were nausea (n = 20), constipation (n = 8), and diarrhea (n = 1). Liraglutide dose reduction was required in one patient due to the adverse reactions. Hypoglycemia was observed in one patient. In the dulaglutide group, nausea (n = 7), diarrhea (n = 6), hepatic dysfunction (n = 3), and hypoglycemia (n = 2) were reported. Gastrointestinal symptoms were reported early after the initiation of treatment but resolved spontaneously.

## Discussion

The present study compared the efficacy and safety of GLP-1 RAs, liraglutide and dulaglutide, in Japanese patients with T2DM in a real-world setting. We used stabilized propensity score-based IPTW in order to reduce selection bias and confounding by observed covariates. In a previously published RCT in Japanese patients, dulaglutide had greater HbA1c-lowering effects than liraglutide^12^. However, the use of other drugs and patient characteristics in that RCT differed from those in real-world clinical practice. The present study included several patients who used more than one oral glucose-lowering agents, unlike previously reported RCT in Japanese patients. In this study using stabilized propensity score-based IPTW, the change in HbA1c level was lower in the liraglutide treatment group at 6 months, but there was no difference in the HbA1c level at 12 months, indicating similar HbA1c-lowering effect by the two agents.

Our study identified three characteristics that were associated with changes in HbA1c over the 12-month treatment period (Table 3). First, baseline HbA1c; patients with high baseline HbA1c levels were more likely to show improved post-treatment HbA1c levels. Next, GLP-1 RA treatment modality; compared with ‘add-on’, the rate of improvement in HbA1c at 12 months was modest in ‘switch’ and ‘reduce’. Similar findings have been reported following liraglutide treatment^13^. In clinical practice, treatment is often switched from dipeptidyl peptidase-4 inhibitors to GLP-1 RAs. In such cases, it is necessary to consider that any improvement in HbA1c after 12-month treatment would be smaller than that after the addition of GLP-1 RAs. Third, initiation of GLP-1 RA treatment during hospitalization was more effective in lowering HbA1c levels than applying the same treatment at the outpatient setting. Although patients who started GLP-1 RA treatment during admission to the hospital had higher baseline HbA1c levels than those who underwent outpatient initiation (9.7% [SD: 1.5] *vs*. 8.3% [SD: 1.4], P < 0.001), their HbA1c levels at 12 months were lower (7.1% [SD: 1.4] *vs*. 7.6% [SD: 1.5], P = 0.032). This result is quite interesting, but it is likely that unmeasured confounding factors and/or differences in follow-up care patterns contributed to this effect, and therefore, further investigation is warranted in the future.

Several studies reported the weight-reducing effects of liraglutide and dulaglutide, with weight loss of 2.3 kg (95% confidence interval [CI] 2.0–2.5) in one study^6^ and 1.46 kg (95% CI 1.25–1.67) in another^14^. However, in the AWARD-6 study using the same two agents, liraglutide (up to 1.8 mg/day) was significantly more effective in reducing body weight than dulaglutide (up to 1.5 mg/week)^11^. Furthermore, in a Japanese phase III clinical study, body weight reduction by dulaglutide was negligible after 26 weeks of treatment^15^. Our results also showed that liraglutide was more effective in reducing body weight than dulaglutide. These results may partly explain why liraglutide is commonly used in obese diabetics in real-world clinical practice.

GLP-1 RAs are reported to help reduce HbA1c, body weight, and insulin dose among insulin users^16–18^. Although there are no studies that directly compared changes in insulin dose after GLP-1 RA initiation (liraglutide *vs.* dulaglutide), our results showed total and bolus insulin dose significantly decreased in liraglutide and dulaglutide, no significant differences were observed between the two agents.

Several reports compared the real-world use of GLP-1 RAs, but most comparisons were restricted to treatment maintenance^19, 20^ and cost-effectiveness^21^. In contrast, our study examined changes in HbA1c levels. A real-world study from Taiwan^22^ showed there was a statistically significant change in HbA1c at 12 months from baseline in each treatment group (dulaglutide: − 1.06% [SD: 1.70] and liraglutide: − 0.83% [SD: 1.61]), with a significant between-group difference in HbA1c reduction of − 0.23% (95% CI − 0.38 to − 0.08%). A study from United States also showed that treatment with dulaglutide significantly reduced HbA1c compared with liraglutide^23^. These results are different from our study, probably due to differences in drug dosage; the dose of dulaglutide in about 40% of the patients of the above study was 1.5 mg/week.

Few studies have investigated the real-world use of GLP-1 RAs in Japanese patients. Although more than 900 patients were enrolled in the JDDM-57 study^10^, HbA1c was analyzed after only 6 months of treatment with GLP-1 RAs. Despite the small sample size, the present study examined the contributing factors to changes in HbA1c induced by GLP-1 RAs in Japanese patients. We anticipate our data to help establish real-world evidence for the role of these factors in GLP-1 RA treatment.

Our study has several limitations. First, the sample size was relatively small, as described earlier, and the study was conducted in patients at a single institution treated by diabetologists; therefore, the results may not be generalizable to the entire population. However, since diabetologists often initiate the administration of GLP-1 RAs in Japan, our findings are considered plausible as real-world data at least in Japan. Second, the plasma glucose data were somewhat unreliable because not all blood samples were collected in a fasting state. However, evaluation of HbA1c, which was the primary endpoint, should be sufficient for the purpose of this study. Third, since liraglutide was the first to be launched in the Japanese market, there was a selection bias that dulaglutide could not be used in patients enrolled early in this study. In addition, the approved maximum dose of liraglutide in Japan under the public health system is 1.8 mg/day, and thus a higher dose of liraglutide was prescribed only in a few patients in the present study. Therefore, it is important to update this study in future investigations to establish more-up-date real-world evidence. Finally, propensity score is only appropriate when the strongly ignorable treatment assignment assumption is satisfied^24^. However, there is no reliable way to test this assumption. Conventionally, consideration is given to C-statistics and propensity score-adjusted estimates of variables, but these also have no standard criteria. In this study, the estimates of the propensity score-adjusted estimates of variables are very close in each group compared to the pre-adjustment, although some variables remained significant, but it is uncertain whether these sufficiently satisfied the strongly ignorable treatment assignment assumption.

In conclusion, we have demonstrated in the present retrospective study the presence of significant differences in the characteristics of patients treated with liraglutide or dulaglutide in a real-world setting, implying that GLP-1 RAs were selected according to individual patient characteristics. Even after reducing the selection bias and confounding using stabilized propensity score-based IPTW between liraglutide and dulaglutide treatments, the effects of these two agents on HbA1c levels after 12 months of treatment were comparable. Our results also suggest that baseline HbA1c level, GLP-1 RA treatment modality, and inpatient initiation of GLP-1 RA treatment may be associated with reduction in HbA1c levels in a real-world setting.

## Patients and methods

### Patients

This retrospective study included patients with T2DM who received outpatient or inpatient care at the Hospital of the University of Occupational and Environmental Health, Japan between September 2010 and August 2019, who underwent GLP-1 RA treatment for the first time, and who continued treatment with liraglutide or dulaglutide for at least 12 months. The following exclusion criteria were applied: patients with type 1 diabetes mellitus, severe infection or serious trauma, and hepatic dysfunction (transaminase level at least threefold higher than the normal upper limit). In addition, we also excluded patients who had used GLP-1 RA previously. This study was approved by the Institutional Ethics Review Committee of the University of Occupational and Environmental Health (approval #H27-186). Informed consent was obtained from the participants, and the study was performed in accordance with the Declaration of Helsinki.

### Biochemical and clinical measurements

We collected patient data, including age, sex, disease duration, body mass index (BMI), arterial blood pressure, presence of diabetic microangiopathy or macroangiopathy, presence of hypertension, dyslipidemia, as well as use of glucose-lowering agents, antihypertensive agents, and antilipidemic medications. Blood and urine samples were collected either in a fasting or non-fasting state. In addition, the levels of plasma glucose, HbA1c, aspartate transaminase (AST), alanine transaminase (ALT), gamma-glutamyl transferase (GGT), and estimated glomerular filtration rate (eGFR) were measured. HbA1c levels (%) were measured using a high-performance liquid chromatography method with a Tosoh HLC-723 G8 analyzer (Tosoh Co., Kyoto, Japan) and expressed in National Glycohemoglobin Standardization Program (NGSP) equivalent values, calculated based on the following equation: HbA1c (NGSP) = HbA1c (Japan Diabetes Society [JDS]) (%) + 0.4%^25^.

Baseline drug adjustments at liraglutide or dulaglutide initiation were categorized as follows: ‘add-on’, when the number of classes of glucose-lowering agents increased; ‘switch’, when the number of classes of glucose-lowering agents remained unchanged; ‘reduce’, when the number of classes of glucose-lowering agents decreased. In addition, if the number of the glucose-lowering agents increased during the 12-month period of GLP-1 RA treatment, the patient was recorded as ‘augmentation’.

### Stabilized propensity score-based IPTW

To adjust for baseline patient characteristics between the two groups, the calculated stabilized propensity scores were weighted using the ‘proportion of patients receiving dulaglutide to all patients/propensity score’ in the dulaglutide group and the ‘proportion of patients receiving liraglutide to all patients/1 − propensity score’ in patients treated with liraglutide as the weighting coefficient on stability^26^. To calculate the stabilized propensity scores, multivariable logistic regression analysis was performed with the concomitant use of dulaglutide as the dependent variable and the following independent variables: age, sex, disease duration, BMI, HbA1c, AST, ALT, eGFR, dyslipidemia, dementia, GLP-1RA treatment modality (add-on, switch or reduce), initiation of GLP-1 RA treatment (outpatient or inpatient), number of oral antidiabetic agent classes, glucose-lowering agents (none, DPP-4 inhibitors, biguanides, SGLT-2 inhibitors, insulin), insulin dose. The area under the curve of the propensity scores model was 0.869 (95% CI 0.818–0.919).

### Statistical analysis

Variables are expressed as mean (standard deviation) or number (%) of patients. Categorical variables were evaluated using the χ^2^ test. Student’s *t*-test or Wilcoxon rank-sum test was employed to compare the two groups, depending on the data distribution pattern. We applied the paired *t*-test or Wilcoxon signed-rank test depending on data distribution to assess changes within the group. Differences between liraglutide and dulaglutide were tested using Student's *t*-test or Wilcoxon rank-sum test. HbA1c target achievement at baseline and 12 months were compared using McNemar’s test. Univariate and multivariate logistic regression analyses were performed to assess the effects of GLP-1 RAs on changes in HbA1c at 12 months. Factors with P < 0.05 on univariate linear regression analysis and the type of GLP-1 RA treatment were entered into multivariate linear regression analysis. Missing data were imputed using the last observation carried forward method, and the results did not differ with or without imputation. Statistical significance was set at P < 0.05. All statistical analyses were performed using SPSS version 25.0 (SPSS Inc., Armonk, NY).

## Supplementary Information

Supplementary Legends.

Supplementary Figure S1.

Supplementary Table S1.

Supplementary Table S2.

## Data Availability

All data generated or analyzed during this study are included in this published article and its Supplementary Information file.

## References

[1] Drucker DJ (1998). Glucagon-like peptides. Diabetes.

[2] Drucker DJ, Nauck MA (2006). The incretin system: Glucagon-like peptide-1 receptor agonists and dipeptidyl peptidase-4 inhibitors in type 2 diabetes. Lancet.

[3] Gutniak M, Orskov C, Holst JJ, Ahrén B, Efendic S (1992). Antidiabetogenic effect of glucagon-like peptide-1 (7–36) amide in normal subjects and patients with diabetes mellitus. N. Engl. J. Med..

[4] Gutzwiller JP (1999). Glucagon-like peptide-1 promotes satiety and reduces food intake in patients with diabetes mellitus type 2. Am. J. Physiol..

[5] Nauck MA, Kemmeries G, Holst JJ, Meier JJ (2011). Rapid tachyphylaxis of the glucagon-like peptide 1-induced deceleration of gastric emptying in humans. Diabetes.

[6] Marso SP (2016). Liraglutide and cardiovascular outcomes in type 2 diabetes. N. Engl. J. Med..

[7] Marso SP (2016). Semaglutide and cardiovascular outcomes in patients with type 2 diabetes. N. Engl. J. Med..

[8] Bethel MA (2018). Cardiovascular outcomes with glucagon-like peptide-1 receptor agonists in patients with type 2 diabetes: A meta-analysis. Lancet Diabetes Endocrinol..

[9] Trujillo JM, Nuffer W, Ellis SL (2015). GLP-1 receptor agonists: A review of head-to-head clinical studies. Ther. Adv. Endocrinol. Metab..

[10] Ishigaki Y (2021). Glucagon-like peptide-1 receptor agonist utilization in type 2 diabetes in Japan: A retrospective database analysis (JDDM 57). Diabetes Ther..

[11] Dungan KM (2014). Once-weekly dulaglutide versus once-daily liraglutide in metformin-treated patients with type 2 diabetes (AWARD-6): A randomised, open-label, phase 3, non-inferiority trial. Lancet.

[12] Odawara M (2016). Once-weekly glucagon-like peptide-1 receptor agonist dulaglutide significantly decreases glycated haemoglobin compared with once-daily liraglutide in Japanese patients with type 2 diabetes: 52 weeks of treatment in a randomized phase III study. Diabetes Obes. Metab..

[13] Simioni N (2018). Predictors of treatment response to liraglutide in type 2 diabetes in a real-world setting. Acta Diabetol..

[14] Gerstein HC (2019). Dulaglutide and cardiovascular outcomes in type 2 diabetes (REWIND): A double-blind, randomised placebo-controlled trial. Lancet.

[15] Onishi Y (2016). Subgroup analysis of phase 3 studies of dulaglutide in Japanese patients with type 2 diabetes. Endocr. J..

[16] Lind M, Jendle J, Torffvit O, Lager I (2012). Glucagon-like peptide 1 (GLP-1) analogue combined with insulin reduces HbA1c and weight with low risk of hypoglycemia and high treatment satisfaction. Prim. Care Diabetes.

[17] Lind M (2015). Liraglutide in people treated for type 2 diabetes with multiple daily insulin injections: Randomised clinical trial (MDI Liraglutide trial). BMJ.

[18] Lee J (2019). Dulaglutide as an add-on to insulin in type 2 diabetes; clinical efficacy and parameters affecting the response in real-world practice. Diabetes Metab. Syndr. Obes..

[19] Norrbacka K (2021). Glucagon-like peptide 1 receptor agonists in type 2 diabetes mellitus: Data from a real-world study in Spain. Diabetes Ther..

[20] Zimner Rapuch S (2021). Treatment patterns and persistence with GLP-1 RA treatments among patients with type 2 diabetes in France: A retrospective cohort analysis. Diabetes Ther..

[21] Dilla T (2017). The cost-effectiveness of dulaglutide versus liraglutide for the treatment of type 2 diabetes mellitus in Spain in patients with BMI 30 kg/m^2^. J. Med. Econ..

[22] Chang KC (2020). Comparative effectiveness of dulaglutide versus liraglutide in Asian type 2 diabetes patients: A multi-institutional cohort study and meta-analysis. Cardiovasc. Diabetol..

[23] Mody R (2019). Adherence, persistence, glycaemic control and costs among patients with type 2 diabetes initiating dulaglutide compared with liraglutide or exenatide once weekly at 12-month follow-up in a real-world setting in the United States. Diabetes Obes. Metab..

[24] Rosenbaum PR, Rubin DB (1983). The central role of the propensity score in observational studies for causal effects. Biometrika.

[25] Committee of the Japan Diabetes Society on the Diagnostic Criteria of Diabetes Mellitus (2010). Report of the committee on the classification and diagnostic criteria of diabetes mellitus. J. Diabetes Investig..

[26] Xu S (2010). Use of stabilized inverse propensity scores as weights to directly estimate relative risk and its confidence intervals. Value Health.

