# Supplementary material for: Real-world effectiveness of liraglutide versus dulaglutide in Japanese patients with type 2 diabetes: a retrospective study
Source: Sci Rep. 2022 Jan 7;12:154. doi: 10.1038/s41598-021-04149-z (PMC8742102; doi:10.1038/s41598-021-04149-z)
Supplement: Supplementary file 1 — Supplementary Legends. [file 41598_2021_4149_MOESM1_ESM.docx]

**Figure S1.** HbAlc levels at baseline and 6 and 12 months of treatment using non-adjusted data.

Data are mean (95% CI). **P<0.01 *vs*. baseline, by Wilcoxon signed-rank test.

HbA1c, glycated hemoglobin.
