# Supplementary figures and images for: Real-world effectiveness of liraglutide versus dulaglutide in Japanese patients with type 2 diabetes: a retrospective study
Source: Sci Rep. 2022 Jan 7;12:154. doi: 10.1038/s41598-021-04149-z (PMC8742102; doi:10.1038/s41598-021-04149-z)

## Slide 1
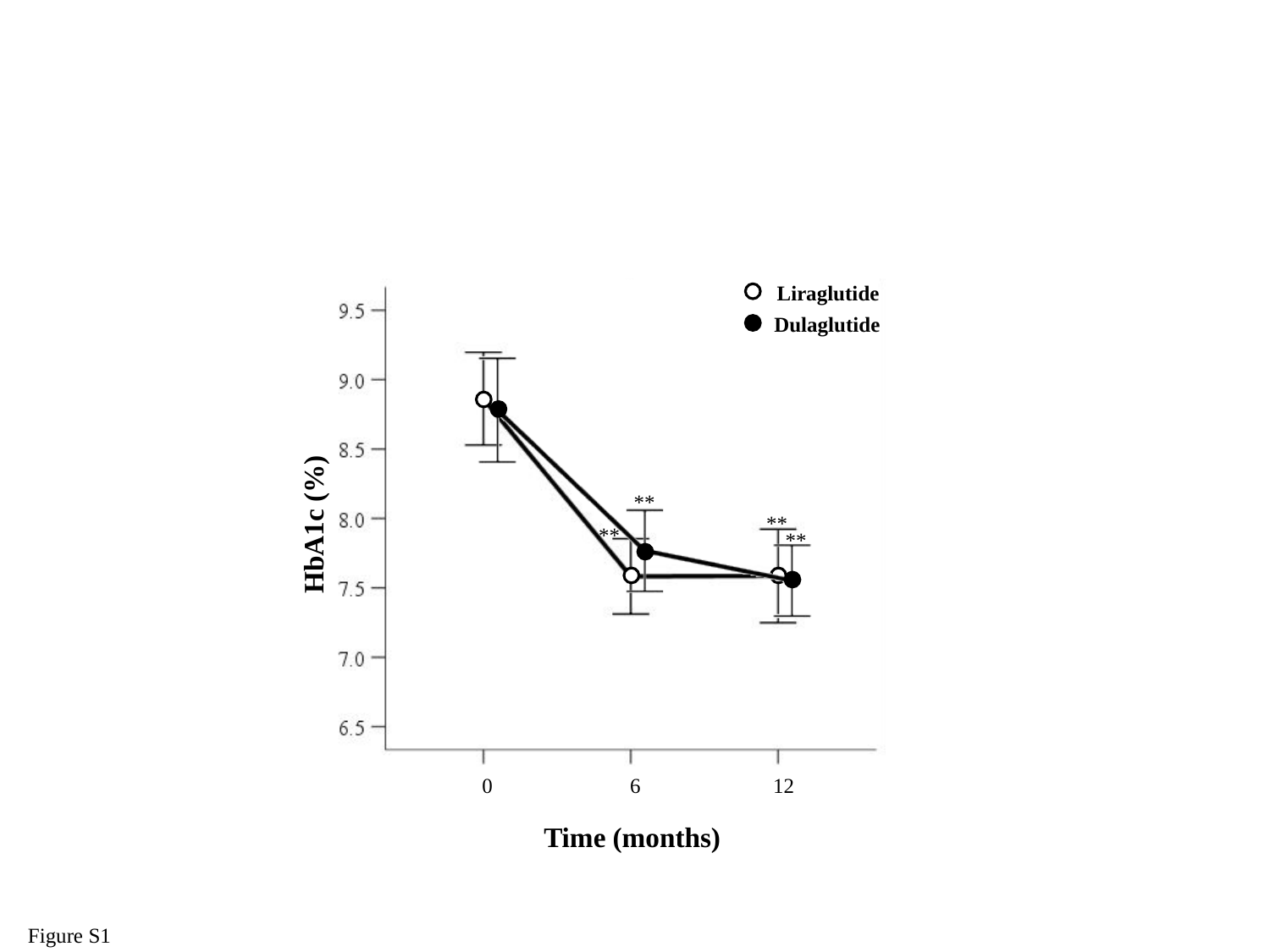

Liraglutide
Dulaglutide
**
HbA1c (%)
**
**
**
0 6 12
Time (months)
Figure S1

Supplement: Supplementary file 2 — Supplementary Figure S1. [file 41598_2021_4149_MOESM2_ESM.pptx]
