# Supplementary material for: Real-world effectiveness of liraglutide versus dulaglutide in Japanese patients with type 2 diabetes: a retrospective study
Source: Sci Rep. 2022 Jan 7;12:154. doi: 10.1038/s41598-021-04149-z (PMC8742102; doi:10.1038/s41598-021-04149-z)
Supplement: Supplementary file 3 — Supplementary Table S1. [file 41598_2021_4149_MOESM3_ESM.docx]

Supplemental Table 1. Results of univariate and multivariate linear regression analyses with change in HbA1c at 12 months of liraglutide treatment as the dependent variable in non-adjusted data.

| Variables | Univariate | | | Multivariate | | |
| --- | --- | --- | --- | --- | --- | --- |
|  | β | 95% CI | P-value | β | 95% CI | P-value |
| Duration of diabetes | 0.247 | 0.010, 0.092 | 0.010 | 0.199 | 0.006, 0.077 | 0.150 |
| PG | -0.263 | -0.012, -0.002 | 0.009 | 0.215 | -0.011, <0.001 | 0.037 |
| HbA1c | -0.524 | -0.741, -0.372 | <0.001 | -0.249 | -0.510, -0.019 | 0.035 |
| ALT | -0.253 | -0.025, -0.003 | 0.012 | -0.105 | -0.016, 0.005 | 0.269 |
| Hypertension | 0.212 | 0.058, 1.894 | 0.037 | 0.012 | -0.764, 0.875 | 0.894 |
| Number of classes of oral glucose-lowering agents | 0.247 | 0.075, 0.665 | 0.015 | 0.116 | -0.096, 0.444 | 0.204 |
| GLP-1RA treatment modality |  |  |  |  |  |  |
| Add-on (reference) |  |  |  |  |  |  |
| Reduced | 0.413 | 0.467, 2.444 | 0.004 | 0.087 | -0.697, 1.311 | 0.545 |
| Switch | 0.277 | -0.013, 2.013 | 0.053 | 0.020 | -0.895, 1.037 | 0.884 |
| Initiation of GLP-1 RA treatment |  |  |  |  |  |  |
| Outpatient (reference) |  |  |  |  |  |  |
| Inpatient | -0.353 | -1.973, -0.590 | ＜0.001 | -0.294 | -1.796, -0.335 | 0.005 |

PG, plasma glucose; HbA1c, glycated hemoglobin; ALT, alanine transaminase, GLP-1 RA, glucagon-like peptide-1 receptor agonist.
