# Supplementary material for: Real-world effectiveness of liraglutide versus dulaglutide in Japanese patients with type 2 diabetes: a retrospective study
Source: Sci Rep. 2022 Jan 7;12:154. doi: 10.1038/s41598-021-04149-z (PMC8742102; doi:10.1038/s41598-021-04149-z)
Supplement: Supplementary file 4 — Supplementary Table S2. [file 41598_2021_4149_MOESM4_ESM.docx]

Supplemental Table 2. Results of univariate and multivariate linear regression analyses with change in HbA1c at 12 months of dulaglutide treatment as the dependent variable in non-adjusted data.

| Variables | Univariate | | | Multivariate | | |
| --- | --- | --- | --- | --- | --- | --- |
|  | β | 95% CI | P-value | β | 95% CI | P-value |
| HbA1c | -0.789 | -1.001, -0.7 | <0.001 | -0.674 | -0.887, -0.570 | <0.001 |
| Dementia | -0.325 | -2.577, -0.553 | 0.003 | 0.063 | -0.361, 0.962 | 0.368 |
| Number of classes of oral glucose-lowering agents | 0.278 | 0.111, 0.841 | 0.011 | 0.114 | -0.214, 0.263 | 0.839 |
| Insulin treatment | 0.231 | 0.054, 1.639 | 0.037 | 0.068 | -0.243, 0.740 | 0.317 |
| GLP-1RA treatment modality |  |  |  |  |  |  |
| Add-on (reference) |  |  |  |  |  |  |
| Reduced | 0.567 | 1.116, 3.343 | <0.001 | 0.318 | 0.522, 1.976 | 0.001 |
| Switch | 0.678 | 1.464, 3.567 | <0.001 | 0.389 | 0.682, 2.206 | <0.001 |
| Initiation of GLP-1 RA treatment |  |  |  |  |  |  |
| Outpatient (reference) |  |  |  |  |  |  |
| Inpatient | -0.474 | -2.606, -1.081 | <0.001 | -0.183 | -1.169, 0.066 | 0.079 |

HbA1c, glycated hemoglobin; GLP-1 RA, glucagon-like peptide-1 receptor agonist.
